# Primary fallopian tube carcinoma: a case report

**DOI:** 10.11604/pamj.2014.18.263.4903

**Published:** 2014-07-30

**Authors:** Houssine Boufettal, Naïma Samouh

**Affiliations:** 1 Departement of gynecology and obstetric « C », Ibn Roshd Universitary Hospital of Casablanca, Morocco

**Keywords:** Adenocarcinoma, Fallopian tube, primitive, radiology, surgery, chemotherapy

## Abstract

Primary cancer of the fallopian tube is very rare and their preoperative diagnosis is difficult due to the lack of specific symptoms. We report a case in a patient aged 42 years, discovered after abdominopelvic mass. Total surgery was performed, followed by platinum-based chemotherapy. The authors report a review of the literature regarding the epidemiology, diagnosis, treatment and prognosis of this cancer.

## Introduction

Primary cancer of the fallopian tube is very rare [1–4]. An average of 20 to 30 new cases are reported each year [5–7]. The incidence of this cancer varies from 0.14 to 1.8% of all gynecological cancers [2]. We report a case of primary tubal adenocarcinoma in a woman aged 42, discovered as a result of an abdominopelvic mass and treated with surgery and chemotherapy. In light of this observation, the authors report a review of the literature on the epidemiology, diagnosis, treatment and prognosis of this cancer.

## Patient and observation

**Figure 1:**
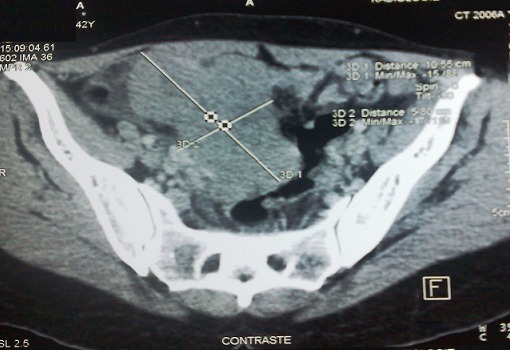
Abdominopelvic CT scan showing a pelvic-abdominal mass with tissue necrosis in places, slightly colored by the injection of contrast

42-year-old woman, single, with no significant personal or family history. She consulted for pelvic pain with pelvic mass that lasting for a month prior toconsultation. The clinical assessment of the patient revealed an abdomino-pelvic mass of hard consistency, measuring three inches, not mobilized from the uterus. On ultrasound of the abdomen and pelvis, the mass appeared tissue and vascular on color Doppler, measuring 112/85 mm. Abdomino-pelvic CT scan showed a pelvi-abdominal tissue necrosis in places, slightly enhanced by injection of contrast, measuring 100/70/60 mm (Figure 1). They were multiple peritoneal nodules with an overdensity of mesenteric fat, pelvic lymph nodes, para-aortic and aorto-caval arteries. The liver and spleen were homogeneous with focal lesion. The CA 125 was increased to 708 IU / mL.

**Figure 2:**
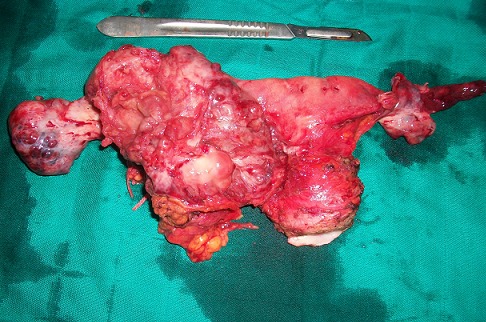
Surgical specimen of hysterectomy showing the right tube. The two ovaries are macroscopically intact

An exploratory laparotomy showed a solid vegetating tumor of the right tube, measuring 100/80 mm. Both ovaries and the contralateral horn were free of any gross lesions. The tumor invaded the bladder and a vermiform appendix. 150 mL of ascites fluid was aspirated. A tumor biopsy of 80/70/20 mm was collected and sent to frozen section for histology. The result of the biopsy showed a poorly differentiated and invasive malignant tumor proliferation. A total hysterectomy with adnexal conservation without appendectomy, with omentectomy and partial resection of the bladder was performed (Figure 2). Parietal and peritoneal biopsies were done. The postoperative course was uneventful. The patient was discharged on day 15 post-op. The pathological diagnosis showed a solid tumor proliferation, whitish, infiltrating, with mesotubular extension, adhering to the front right side of the uterus and measuring 12 centimeters in diameter. The microscopic aspect was consistent with adenocarcinoma, poorly differentiated, infiltrating, necrotic, with mesotubular and meso appendix extension. A chest radiograph was normal. No recurrence was noted at 6 months post-op.

## Discussion

Primary cancer of the fallopian tube is a rare gynecological cancer [1, 2]. An average of 20 to 30 new cases reported annually [3, 4]. The rarity of this cancer is due to the fallopian tube low oncogenic potential, in contrast to the vulnerability of the organ to infection. It is generally of poor prognosis [5–7]. It was reported by Renaud in 1847 [1, 8], but it is Orthmann [2] in 1888 who reported the first documented description. The incidence of this cancer varies from 0.14 to 1.8% of all gynecological cancers [2, 4]. The average annual incidence of the cancer of the fallopian tube in the United States is 3.6 per million women per year [4]. In Finland the incidence is 5.4 per million between 1993 and 1997 [3]. The true incidence is certainly underestimated. Indeed, the diagnosis can be made wrongly as ovarian cancer, it is during the initial surgery or during the examination and pathology. Cancer of the fallopian tube occurs most often after the fourth decade of life, with an average age of 62 years (17-88 years) [2, 9, 10]. No case is reported in childhood. The age of our patient is 42 years younger than the average age reported in the literature [2–4]. The consultation period for patients with cancer of the fallopian tube is shorter than that of patients with ovarian cancer, because of abdominal pain secondary to distention of the fallopian tubes [4, 11]. Abdominal pain, vaginal bleeding, hydrohematorrhea, impaired general condition and palpation of a pelvic mass are the most common symptoms during the primary cancer of the fallopian tube [11].

Adominopelvic ultrasound often shows a tissue mass, heterogeneous, with different sizes, retro or latero uterine. Abdominopelvic CT scan shows a latero uterine pelvic tissue damage. The lesion is enhanced after injection of contrast. The uterus is usually of normal size [1–5]. However, diagnosis is virtually difficult before laparotomy. The concept of pelvic pain in the history of the disease, due to distension of the tubes, combined with latero uterine pelvic mass on imaging should suspect a tumor of the fallopian tube. Diagnosis is rarely made preoperatively. Intraoperative diagnosis itself is not possible in 50% of cases due to tumor extension [6]. And even when the tumor is resected, it is possible not to report the diagnosis of cancer on pathological examination, and to report it as an ovarian cancer [4, 6].

The disease is often advanced, but it is long confined to the peritoneal cavity. The risk of bilateral disease is 10 to 27% of cases [4].

Because of the rarity of this cancer, there is no consensus on diagnosis support and treatment. Some groups recommend to complement the surgery with pelvic radiotherapy, while others argue that a combination with cisplatin chemotherapy, improves short-term survival [7].

Surgery is the treatment of choice for cancer of the fallopian tube. The surgical principles are the same as those of ovarian cancer. The cytoreductive surgery taking as much as possible of the tumor is warranted in patients with advanced disease. However, given the strong tendency to lymphatic spread of the tumor, a systematic pelvic and para-aortic lymphadenectomy should be preferred to sample lymph nodes [4, 6, 11]. The left lateroaortique chain above is the most frequent mesenteric involvement. Isolated pelvic invasion is rare. Thus, lymphadenectomy should absolutely include not only the pelvic lymph nodes, but also and especially para-aortic lymph nodes on the bottom edge of the left renal vein [11].

Given the mode of lymphatic and hematogenous spread of this type of cancer, chemotherapy is warranted, especially in the early stages of the disease. The gold standard is the combination platinum-taxane, as in epithelial tumors of the ovary [2]. The response rate of cisplatin-based chemotherapy is 53 to 92%. By cons there are few data on the response to this chemotherapy in advanced stages.

Pectasides et al. [2], in a series of 64 patients, obtained an overall response rate of 93% (best in all publications) for a combination of platinum-based chemotherapy with carboplatin and paclitaxel. Peters et al. [12] reported a complete response rate of 75% in a study of 46 patients. Similarly, in a series of 45 patients treated with cisplatin, Gadducci et al. [5], reported rates of complete and partial response of 64.4% and 17.8% respectively, with a five-year survival of 56% for complete response and only 21 months for partial response.

## Conclusion

Primary tubal cancer is rare, of unknown etiology and sometimes mistaken for uterine or ovarian pathology. The clinical signs are rarely present in full. The diagnosis is rarely made preoperatively or on histology. Because of its histopathological similarities with ovarian cancer, its treatment is similar to that of the latter.
